# Depression and Anxiety as Predictors of Quality of Life in Osteoarthritis Patients

**DOI:** 10.7759/cureus.93872

**Published:** 2025-10-05

**Authors:** Yashar Mashayekhi, Sara Baba-Aissa, Amanuel Kefyalew Assefa, Francis T Mutamba, Aamir Mohamed Nur, Zuhaib Y Shahid, Naheemat Mofolasayo Salimon, Ahmad Habahbeh, Niamat Ali, Ibrahim M Shandi, Racha Al Niazi, Fatima Habib

**Affiliations:** 1 Medicine, University Hospitals of Leicester NHS Foundation Trust, Leicester, GBR; 2 General Internal Medicine, Leicester Royal Infirmary, Leicester, GBR; 3 Orthopaedics and Trauma, University Hospitals of Leicester NHS Foundation Trust, Leicester, GBR; 4 Diabetes and Endocrinology, University Hospitals Birmingham, Birmingham, GBR; 5 Haematology, University Hospitals of Leicester NHS Foundation Trust, Leicester, GBR; 6 Trauma and Orthopedics, Maidstone and Tunbridge Wells NHS Trust, Tunbridge Wells, GBR; 7 Orthopaedics, Teesside University, Middlesbrough, GBR; 8 Orthopaedics, Marienhospital Wesel, Academic Teaching Hospital of the University of Münster, Wesel, DEU; 9 Orthopaedic Surgery, Lahore General Hospital, Lahore, PAK; 10 Pharmacology/Internal Medicine, Ras Al Khaimah Medical and Health Sciences University, Dubai, ARE; 11 Orthodontics, Dubai Health, Dubai, ARE; 12 Medicine, Rai Medical College Sargodha, Sargodha, PAK

**Keywords:** anxiety, depression, hads, osteoarthritis, quality of life, sf-36

## Abstract

Background: Osteoarthritis (OA) is among the most common chronic joint disorders, which not only causes pain and disability, but also adversely impacts the psychological health and the quality of life (QoL). Depression and anxiety are commonly reported in OA patients, but their use as predictors of QoL has not been fully explored in Pakistan. The purpose of this study was to investigate whether depression and anxiety have a predictive effect on QoL in OA patients.

Methods: A cross-sectional study was conducted between July and August 2025 among 300 adult OA patients recruited through community health centers, outpatient clinics in Sargodha and Lahore, Pakistan, and orthopaedic departments. The tools used to collect data were a structured questionnaire, consisting of the Hospital Anxiety and Depression Scale (HADS) and the Short Form-36 Health Survey (SF-36). Descriptive statistics, Pearson correlation, independent t-tests, one-way ANOVA, chi-square tests, and multiple linear regression were used to evaluate relationships between psychological and QoL.

Findings: The study population consisted primarily of female patients (N = 240, 80%), with half being older than 65 years (N = 150, 50%). There was a significant correlation between anxiety and depression with lower QoL (r = -0.356 and r = -0.482, respectively; p < 0.001). Women were found to have average anxiety and depression and a low QoL score relative to that of men (p < 0.001). Older age was linked to greater depression and anxiety and less QoL (p < 0.001). Regression analysis revealed a significant predictor of poor QoL as depression (β = -0.245, p = 0.001), anxiety (β = -0.145, p = 0.003), older age, female gender, longer duration of disease, location of OA, and current treatment.

Conclusion: Depression and anxiety became powerful predictors of poor QoL among OA patients, and depression had a more substantial effect. The results emphasise the need to integrate psychological evaluation and care into the regular OA treatment to enhance patient outcomes. Both physical and mental health care should be given on a holistic basis to improve the QoL of this population.

## Introduction

Osteoarthritis (OA) is an inflammatory degenerative condition of the joints, caused by multiple factors, characterised by tissue and cartilage degradation and inflammation, which leads to pain. Currently, there is no disease-modifying treatment available [1]. It is highly prevalent, with obesity and joint injury being significant modifiable risks. The estimate suggests an overall prevalence of 14.8%, with higher rates in women and older people, where obesity was identified as a risk factor, and physical activity and vitamin C use were found to be protective factors [2,3].

In addition to physical symptoms, OA also has a significant impact on psychological well-being. Although some patients develop acceptance as a coping strategy, normalisation of pain leads to a decrease in interest in everyday life and impairs life quality [4]. Approximately one in every five OA patients has reported anxiety and depression; it is associated with greater pain, increased utilisation of healthcare services, and worse performance. Although the prevalence compared to those without OA is unclear, holistic management that addresses psychological comorbidities should be encouraged to enhance quality of life (QoL) [5,6].

Women with knee OA experience increased levels of anxiety and depression and a poorer QoL than women without OA. These findings reinforce the importance of integrating pharmacological, psychological, and social support during OA management [7]. OA also reduces functional capacity and worsens pain, and lower education is associated with poorer perceived QoL, where sociodemographic factors appear to moderate OA outcomes [8].

Finally, knee OA is closely linked to poorer mental health, such as depressive mood, psychological distress, or suicidal ideation, and decreased health-related quality of life (HRQoL) in older adults and people of middle age [9]. OA leads to a decrease in QoL, affecting physical activity, mental health, and psychosocial and daily activities in elderly patients. General and age-specific QoL aspects are influential and should be assessed to manage them effectively [10,11].

The purpose of the current study is to examine depression and anxiety as predictors of QoL among OA patients. By determining the magnitude of the contribution of identified psychological variables to QoL, this study aims to highlight the necessity of establishing integrated strategies that combine the physical and psychological dimensions of OA management.

Rationale

OA is a chronic disease that can have a profound impact on the physical and mental health of patients. Although the physical manifestations of OA, including pain, stiffness, and impaired mobility, have been widely acknowledged, the psychological factors, especially depression and anxiety symptoms, are equally significant in influencing the QoL of patients. In different countries, the work is already available on how mental well-being influences the general well-being of OA patients, who experience depression and anxiety that aggravate the experience of pain and constrain their daily activities. Nevertheless, despite the existing studies, there is little evidence about the Pakistani context, where culture, social, and health aspects might determine both the rates of psychological distress and its effects on the QoL.

Therefore, exploration of depression and anxiety as determinants of QoL in Pakistani OA patients is essential in filling the contextual gap. Exploring the role of psychological factors in shaping the overall burden of living with OA can assist medical professionals in creating more holistic treatment regimens that cater to its physical and mental health aspects. The limited scope of the intervention proposed in this study is expected to yield locally relevant findings that will be directly applicable to clinical practice in Pakistan, ensuring that any intervention meets the local context and challenges of the patient population at stake.

Primary objective

The primary objective is to investigate whether depression and anxiety predict the QoL in patients with OA.

Secondary objectives

The secondary objectives are to determine the prevalence of depression and anxiety among Pakistani patients with OA, to examine associations between these psychological factors and specific QoL domains (physical, social, and emotional), and to explore demographic and clinical patterns related to depression, anxiety, and QoL.

## Materials and methods

Methodology

This study utilised a cross-sectional design to determine the relationship between depression, anxiety, and QoL among people diagnosed with OA. The data were gathered in Sargodha and Lahore, Pakistan, from relatively large communities and adults aged 18 and over with OA who were undergoing treatment or follow-up. Participants were sampled through a combination of community and health centres, as well as orthopaedic clinics and outpatient departments in various hospitals. These recruitment sites were government hospitals and community-based clinics that focus primarily on low-income patients, older adults, and those with limited access to specialised care. This approach enabled a diverse group of participants, representing various demographics, including different age ranges, genders, educational backgrounds, and socioeconomic statuses, despite certain groups being overrepresented, such as older adults and women.

The standardised and validated questionnaires were used in assessing depression, anxiety, and QoL. Further demographic and clinical information, such as age, gender, level of education, comorbidities, duration of OA, and current treatment, was also measured using other interview-based items. This information was applied to analyse any potential correlations between psychological manifestations and QoL outcomes.

Sample size and technique

The required sample size was estimated using a standard formula for prevalence studies, assuming a 95% confidence level, 5% margin of error, and an expected proportion of 0.50 to yield the maximum possible sample size [12]. This calculation suggested a sample size of 385 participants. However, due to feasibility and logistical constraints, the final study included 300 participants.

A convenience sample was used to recruit the participants. Participants who met the eligibility criteria were recruited. Data collectors approached patients in the waiting area, informed them about the research goals, and then provided them with information about the study and obtained informed consent before enrollment. A total of 300 participants volunteered to take part in the survey and completed it. Participants did not receive monetary or material incentives. As convenience sampling was used, reproducibility across different populations may be limited, which we acknowledge as a methodological constraint.

Participant selection criteria

In this study, the participants consisted of adult individuals 18 years of age or older with physician-diagnosed OA, confirmed by treating physicians through clinical examination and medical records, who were undergoing treatment or follow-up in outpatient facilities, privately owned clinics, or community health centres in the Islamabad and Rawalpindi regions. Only those who could respond to the questionnaires and give informed consent were included. Patients with other types of arthritis (i.e., with rheumatoid arthritis, gout, and psoriatic arthritis) and if they had any severe psychiatric conditions, such as schizophrenia or bipolar disorder, that would complicate the determination of the depression and anxiety items were excluded. People with cognitive disabilities or neurological disorders like dementia or stroke that might compromise their competency to take the survey were also excluded. Additionally, patients who refused to participate or failed to provide informed consent were excluded from the study.

Research tools and measures

To collect data, a questionnaire was designed in three parts: demographic data, evaluation of depression and anxiety, and evaluation of QoL. They were administered using validated and standardised scales, together with a brief demographic form created by the researchers. All instruments were administered in their original English versions, without any cultural or linguistic modifications. While this minimised variability in interpretation, it may limit applicability in non-English-speaking groups.

Demographic information

The first section of the questionnaire was meant to gather demographic data and establish the background of the research participants. Age, gender, marital status, level of education, occupation, and duration of OA were taken into consideration. Data regarding comorbidities and treatment conditions were also collected. These variables were deemed relevant in determining potential contributors to psychological symptoms and the general QoL. The gathering of this information provided a more defined picture of the study population and facilitated a simpler subgroup analysis.

Hospital Anxiety and Depression Scale (HADS)

The HADS was utilised for measuring anxiety and depressive symptoms in patients with OA. The instrument is divided into two parts, each comprising seven questions. One part assesses anxiety, and the other assesses depression. The responses are graded using a four-point Likert scale of 0-3, where the higher the rating, the worse the anxiety or depression symptoms. The scale was first developed by Zigmond and Snaith in 1983; it has been proven to have high reliability. The reliability analysis indicated that Cronbach alpha coefficients of the anxiety and depression subscales were 0.83 and 0.82, respectively, of HADS [13]. Permission to use the Hospital Anxiety and Depression Scale (HADS) was obtained from Mapi Research Trust (Work Order No. 2511678) under a Master User License Agreement.

Short Form-36 Health Survey (SF-36)

The SF-36 was used to measure the QoL among patients with OA. Introduced by Ware and Sherbourne in 1992, the instrument is extensively used to measure HRQoL. It consists of 36 items that represent eight health domains: physical functioning, role limitations due to physical health issues, bodily pain, general health perceptions, vitality, social functioning, role limitations due to emotional health issues, and mental health. Each of the given domains is rated independently on a scale of 0-100, with higher scores reflecting higher progress in health levels and QoL. The SF-36 is highly reliable across different populations, with Cronbach's alpha values ranging from 0.78 to 0.93 in most subscales, indicating its internal consistency. It was designed to assess both physical and psychological aspects of well-being simultaneously, making it especially applicable to samples with chronic diseases, such as OA [14]. The questionnaire was freely available for use in research. The original SF-36 survey remained unchanged. The formulation of SF-36 is credited to the RAND Corporation. It is applied according to RAND terms regarding the SF-36 tool [15].

Procedure

The participants were identified through outpatient services at public hospitals, privately owned orthopaedic clinics, and community health centres in Sargodha and Lahore. Data were collected over two months (July-August 2025) during routine patient visits to the clinic. Eligible participants were identified in waiting areas and informed of the purpose and characteristics of the studies in clear and straightforward terms to ensure they understood everything clearly. Informed consent was obtained in a written manner.

The individuals who consented to participate received the study questionnaire comprising both the demographic form, the HADS, and the SF-36. Participants could fill out the questionnaire independently or with the assistance of trained research personnel, depending on their literacy level and personal preference. Privacy and confidentiality were considered. Personal data, such as names, was removed, and all answers were treated anonymously. It employed an ethical and culturally sensitive methodology, allowing respondents to answer questions with considerable freedom and ease.

Analytical approach

Data analysis was performed using IBM SPSS Statistics for Windows, Version 26 (Released 2019; IBM Corp., Armonk, New York, United States). Descriptive statistics were used to describe the demographic and clinical characteristics of the study population (N = 300) in terms of frequency, percentage, mean, and standard deviation. None of the missing data was imputed before performing the analysis, and incomplete responses were removed pairwise to avoid compromising data accuracy without cycling unnecessarily. Pearson correlation analysis was used to determine the correlation of the anxiety, depression, and QoL (SF-36) scores. One-way analysis of variance (ANOVA) was used to compare anxiety, depression, and QoL across sites of OA. Multiple linear regressions were chosen to determine significant clinical and demographic predictors of QoL. In addition, a chi-square test of independence was used to ascertain the correlation between the location of OA and the existing treatment mode. All analyses were two-sided, and significance was considered p < 0.05.

Ethical protocols

The study was conducted in accordance with the ethical requirements for research involving human subjects. The IRB of the Rai Medical College Sargodha, RMCS/IRB/2025/034, reviewed and approved the research protocol. No ethical issues were raised or violated during the study, as it adhered to the principles of respect for persons, beneficence, and confidentiality. All participants were informed of the research objectives, methods, potential risks, and benefits in a language they could understand before the data collection process began. The involvement was voluntary, and written informed consent was taken before enrolment.

The participants were informed that they could withdraw from the research at any time without any consequences to their future care. No personal data was collected, and all information underwent an anonymisation procedure at the moment of entry, processing, and analysis to ensure confidentiality. The questionnaires were coded as complete cases, and any with incomplete responses on key activities were removed before final analysis to maintain the quality and precision of the outcomes. The dignity, privacy, and well-being of all the participants were addressed at all levels of the research process.

## Results

Table 1 presents the demographic and clinical profile of the study participants (N = 300). Half of the sample (N = 150, 50%) was aged 65 years and above, a smaller proportion of 60-64 years (N = 40, 13%), 50-59 years (N = 35, 12%), 40-49 years (N = 30, 10%), and 30-39 years (N = 25, 8%), and 18-29 years (N = 20, 7%). Most of them were female (N = 240, 80%) as opposed to male participants (N = 60, 20%). Of the participants, 124 (41%) were married, 106 (35%) were single, and a few were widowed (N = 59, 20%) or divorced/separated (N = 11, 4%). Regarding education, 99 (33%) had primary education, 87 (29%) had a high school education, 67 (22%) had no formal education, 34 (11%) held a bachelor's degree, and 13 (4%) held a master's degree. In terms of occupation, 106 (35%), 92 (31%), 62 (21%), and 40 (13%) were unemployed, retired, employed, and students, respectively. OA was most prevalent in the hip (N=104, 35%), hand (N=63, 21%), knee (N=51, 17%), foot/ankle (N=29, 10%), shoulder (N=23, 8%), spine (N=20, 7%), and elbow (N=10, 3%). The time of OA was 1-5 years in 101 (34%), <1 year in 91 (30%), 6-10 years in 70 (23%), and >10 years in 38 (13%). The most common comorbidities were cardiovascular disease (N = 99, 33%), diabetes (N = 65, 22%), hypertension (N = 58, 19%), respiratory disease (N = 47, 16%), and gastrointestinal disease (N = 21, 7%). Only 10 (3%) did not report a comorbidity. Amongst the management, the largest were intra-articular injections (N = 103, 34%), pain medications (N = 80, 27%), physiotherapy/exercise (N = 60, 20%), no treatment (N = 37, 12%), and surgery (N = 20, 7%).

**Table 1:** Demographic Profile of Participants (N = 300) f: frequency; %: percentage; values are presented as N (%); N: 300; no statistical comparisons were performed for demographic variables in this table.

Variable	f (N)	%
Age		
18–29 years	20	7
30–39 years	25	8
40–49 years	30	10
50–59 years	35	12
60–64 years	40	13
65 years & above	150	50
Gender	-	-
Male	60	20
Female	240	80
Marital status	-	-
Single	106	35
Married	124	41
Widowed	59	20
Divorced/separated	11	4
Educational level	-	-
No formal education	67	22
Primary school	99	33
Higher school	87	29
Bachelor's degree	34	11
Master's degree	13	4
Occupation	-	-
Student	40	13
Employed	62	21
Unemployed	106	35
Retired	92	31
Site of osteoarthritis	-	-
Knee	51	17
Hip	104	35
Hand (fingers, thumb)	63	21
Foot/ankle	29	10
Shoulder	23	8
Elbow	10	3
Spine (neck/back)	20	7
Duration of osteoarthritis diagnosis	-	-
< 1 year	91	30
1-5 years	101	34
6-10 years	70	23
>10 years	38	13
Comorbid conditions	-	-
Hypertension	58	19
Diabetes	65	22
Cardiovascular disease	99	33
Respiratory disease	47	16
Gastrointestinal disease	21	7
None	10	3
Current treatment for osteoarthritis	-	-
No treatment	37	12
Pain medications (NSAIDs, analgesics)	80	27
Intra-articular injections	103	34
Physiotherapy /Exercise program	60	20
Surgery (past or planned)	20	7

Table 2 presents the results of the normality test for the study variables (N = 300). The Kolmogorov-Smirnov test (p > 0.200 on all measures) and the Shapiro-Wilk test (HADS-A: p = 0.712; HADS-D: p = 0.648; SF-36: p = 0.835) demonstrated that the data on the three measures were usually distributed.

**Table 2:** Normality Testing for Study Variables (N = 300) The Kolmogorov–Smirnov test used Lilliefors significance correction; df: degree of freedom; values are presented as test statistic (df = 300), with corresponding p-values; A p-value > 0.01 was considered statistically significant, indicating normal distribution; N = 300.

Variable	Kolmogorov–Smirnov Statistic	df	p	Shapiro–Wilk Statistic	df	p
Hospital Anxiety and Depression Scale–Anxiety subscale (HADS-A) [13]	0.035	300	>0.200	0.997	300	0.712
Hospital Anxiety and Depression Scale–Depression subscale (HADS-D) [13]	0.032	300	>0.200	0.996	300	0.648
SF-36 [14]	0.029	300	>0.200	0.998	300	0.835

Table 3 shows that anxiety, depression, and QoL are significantly correlated in patients with OA (N = 300). The positive relationship was between anxiety and depression (r = 0.412, p < 0.001), and the negative relationship between anxiety and QoL (r = -0.356, p<0.001). On the same note, depression and QoL had a negative relationship (r = -0.482, p = 0.001). These results confirm that anxiety and depressive levels are related to low QoL in this population of patients.

**Table 3:** Pearson Correlations Among Anxiety, Depression, and Quality of Life in Osteoarthritis Patients (N = 300) Values represent Pearson correlation coefficients (r) between continuous variables, with corresponding t-test statistics and significance values; df: 298. Pearson’s correlation test was conducted; p < .001** (2-tailed) was considered statistically significant and is denoted with double asterisks (**).

Variables	Hospital Anxiety and Depression Scale–Anxiety subscale	Hospital Anxiety and Depression Scale–Depression subscale	Short Form Health Survey
Hospital Anxiety and Depression Scale–Anxiety subscale [13]	-	Pearson’s r = 0.41, t(298) = 7.98, p<0.001^**^	Pearson’s r = –0.36, t(298) = –6.76, p<0.001^**^
Hospital Anxiety and Depression Scale–Depression subscale [13]	-	–	Pearson’s r = –0.48, t(298) = –9.71, p< 0.001^**^
Short Form Health Survey [14]	-	-	-

Table 4 demonstrates substantial gender disparities in anxiety, depression, and QoL among patients with OA (N = 300). Female patients scored higher on anxiety (M = 19.40, SD = 2.10) than male patients (M = 17.80, SD = 2.20; t = -5.10, p < 0.001, d = 0.76), and depression scores (M = 19.70, SD = 2.05) were also statistically higher than that registered by male patients (M = 18.30, SD = 2.25; t = -4.20). Male patients, in turn, had a higher QoL score (M = 88.50, SD = 4.80) than female patients (M = 85.40, SD = 5.10; t = 3.90, p < 0.001, d = 0.62). These results demonstrate that the psychological distress and the QoL of female patients were significantly higher in comparison to those of male patients.

**Table 4:** Gender Differences in Anxiety, Depression, and Quality of Life in Osteoarthritis Patients (N = 300) Values are presented as Mean ± Standard Deviation; independent sample t-tests were conducted to compare participants of both genders; reported statistics include t-values with degrees of freedom (t(298)), p-values, 95% confidence intervals (CIs), and effect sizes (Cohen's d); group sizes are shown as N (%); a p-value < 0.01** was considered statistically significant, N = 300.

Variable	Male (N=60;20%) M±S.D	Female (N=240;80%) M±S.D	Independent samples t	p	Cl 95% LL	UL	Cohen’s D
Hospital Anxiety and Depression Scale–Anxiety subscale (HADS-A) [13]	17.80±2.20	19.40±2.10	–5.10	<0.001^**^	–2.21	–0.99	0.76
Hospital Anxiety and Depression Scale–Depression subscale (HADS-D) [13]	18.30±2.25	19.70±2.05	–4.20	<0.001^**^	–2.05	–0.75	0.67
Short Form Health Survey (SF-36) [14]	88.50±4.80	85.40±5.10	3.90	<0.001^**^	1.54	4.66	0.62

Table 5 indicates that anxiety, depression, and QoL in patients with OA significantly vary between different ages (N = 300). The score on the anxiety question remained higher as one aged, with a low setting of M = 15.2 (SD = 2.0) in 18 to 29-year-olds, rising to M = 20.1 (SD = 2.4) in those aged 65 years and above (F(5, 294) = 24.9, p < 0.001). The same pattern occurred in the case of depression, where the mean scores increased from 15.0 (SD = 2.1) to 20.2 (SD = 2.5) in the lowest and highest age groups, respectively (F(5,294) = 26.4, p < 0.001, η² = 0.31). On the contrary, the QoL (SF-36) scores showed a declining trend with younger participants registering a higher QoL (M = 92.5, SD = 4.5) than the oldest population (M = 82.5, SD = 5.3; F(5,294) = 24.8, p < 0.001, η 2 = 0.30). These findings suggest that there is a certain tendency for deterioration of psychological distress and a decline in QoL in old age.

**Table 5:** Means, Standard Deviations, and ANOVA Results for Anxiety, Depression, and Quality of Life Across Age Groups (N = 300) Data are presented as mean ± standard deviation (M ± SD); group sizes are shown as N (%). One-way ANOVA (F(5, 294)) was conducted to examine the effect of age group. Higher scores on HADS-A and HADS-D indicate greater anxiety and depression. Higher SF-36 scores indicate better quality of life; all comparisons were significant at p < 0.001**; η² represents the partial eta-squared effect size.

Variable	18–29 years (N=20;7%) M±S.D	30–39 years (N=25;8%) M±S.D	40–49 years (N=30;10%) M±S.D	50–59 years (N=35;12%) M±S.D	60–64 years (N=40;13%) M±S.D	65+ years (N=150;50%) M±S.D	One-way ANOVAF(5, 294)	p	η²
Hospital Anxiety and Depression Scale–Anxiety subscale (HADS-A) [13]	15.2±2.0	16.0±2.1	17.1±2.2	18.4±2.3	19.2±2.3	20.1±2.4	24.9	< 0.001^**^	0.30
Hospital Anxiety and Depression Scale–Depression subscale (HADS-D) [13]	15.0±2.1	16.1±2.2	17.4±2.2	18.6±2.3	19.3±2.4	20.2±2.5	26.4	< 0.001^**^	0.31
Short Form Health Survey (SF-36) [14]	92.5±4.5	90.8±4.7	88.6±4.9	86.2±5.1	84.9±5.2	82.5±5.3	24.8	< 0.001^**^	0.30

Table 6 presents differences among anxiety, depression, and QoL among OA sites (N = 300). The scores of anxiety were not significantly different among sites (F(6,293) = 1.54, p = 0.17, 0.031). Depression scores, however, responded significantly to the OA site (F (6,293) = 2.82, p = 0.010, 0.055), whereas the mean score was the highest among OA patients with elbow (M = 20.70, SD = 1.95), and spine (M = 19.95, SD = 2.16) entailments. There was no significant difference between sites in QoL (SF-36) scores (F (6,293) = 1.70, p = 0.12). These results indicate that depression is affected by the OA site, whereas the effect of such site on anxiety or the QoL is not provided.

**Table 6:** One-way ANOVA of Osteoarthritis Site Differences on Anxiety, Depression, and Quality of Life (N = 300) Data are presented as mean ± standard deviation (M ± SD); group sizes are shown as N (%); one-way ANOVA (F(6, 293)) was conducted to compare the site of osteoarthritis on Hospital Anxiety and Depression Scale–Anxiety subscale (HADS-A), Hospital Anxiety and Depression Scale–Depression subscale (HADS-D), and Short Form Health Survey (SF-36) scores; a p-value < 0.01** was considered statistically significant; η² represents the partial eta-squared effect size.

Variable	Knee (N = 51; 17%) M ± SD	Hip (N = 104; 35%) M ± SD	Hand (N = 63; 21%) M ± SD	Foot/Ankle (N = 29; 10%) M ± SD	Shoulder (N = 23; 8%) M ± SD	Elbow (N = 10; 3%) M ± SD	Spine (N = 20; 7%) M ± SD	One-way ANOVA F(6, 293)	p	η²
Hospital Anxiety and Depression Scale–Anxiety subscale (HADS-A) [13]	18.53 ± 2.48	18.88 ± 2.54	19.05 ± 2.07	18.83 ± 2.25	19.61 ± 2.73	19.40 ± 2.50	18.60 ± 2.04	1.54	0.17	0.031
Hospital Anxiety and Depression Scale–Depression subscale (HADS-D) [13]	19.29 ± 2.50	19.10 ± 2.41	19.22 ± 2.48	17.97 ± 1.92	18.61 ± 2.71	20.70 ± 1.95	19.95 ± 2.16	2.82	0.010^**^	0.055
Short Form Health Survey (SF-36) [14]	84.71 ± 5.30	86.53 ± 5.33	86.84 ± 5.77	87.45 ± 6.91	87.30 ± 5.12	87.00 ± 4.03	84.85 ± 5.68	1.70	0.12	0.034

Table 7 presents the regression model, where the QoL (as measured by the SF-36) is predicted using demographic and clinical factors (N = 300). Effective predictors of low QoL were the presence of high anxiety (B -0.320, p 0.003), high depression (B -0.540, p < 0.001), older age (B -0.420, p 0.004), female gender (B -2.350, p 0.009), site of OA (B -0.310, p 0.014), and longer duration of diagnosis (B -0.280). Depression (beta = -0.245) and anxiety (beta = -0.145) had the most significant effect. In general, the findings demonstrate that the depressive symptoms and clinical-demographic conditions were substantial contributors to impaired QoL among OA patients.

**Table 7:** Multiple Regression Predicting Quality of Life (SF-36) from Clinical and Demographic Factors (N = 300) Multiple linear regression analysis was conducted to identify predictors of Short Form Health Survey (SF-36) scores; reported statistics include unstandardised coefficients (B), 95% confidence intervals (CIs), standard error (SE), standardised beta coefficients (β), t-values, and p-values; a p-value < 0.01** was considered statistically significant, N = 300.

Predictor	B	S.E	β	t (Regression coefficient test)	p	(95% CI) LL	UL
Constant (Short Form Health Survey) [14]	102.345	5.820	—	17.580	<0.001^**^	90.900	113.790
Hospital Anxiety and Depression Scale–Anxiety subscale (HADS-A) [13]	-0.320	0.105	-0.145	-3.048	0.003^**^	-0.527	-0.113
Hospital Anxiety and Depression Scale–Depression subscale (HADS-D) [13]	-0.540	0.110	-0.245	-4.909	<0.001^**^	-0.756	-0.324
Age	-0.420	0.145	-0.115	-2.897	0.004^**^	-0.706	-0.134
Gender	-2.350	0.890	-0.125	-2.642	0.009^**^	-4.100	-0.600
Site of Osteoarthritis	-0.310	0.125	-0.095	-2.480	0.014^*^	-0.555	-0.065
Duration of Osteoarthritis diagnosis	-0.280	0.115	-0.090	-2.435	0.016^*^	-0.506	-0.054
Current treatment for Osteoarthritis	-1.420	0.480	-0.135	-2.958	0.003^**^	-2.360	-0.480

**Figure 1:**
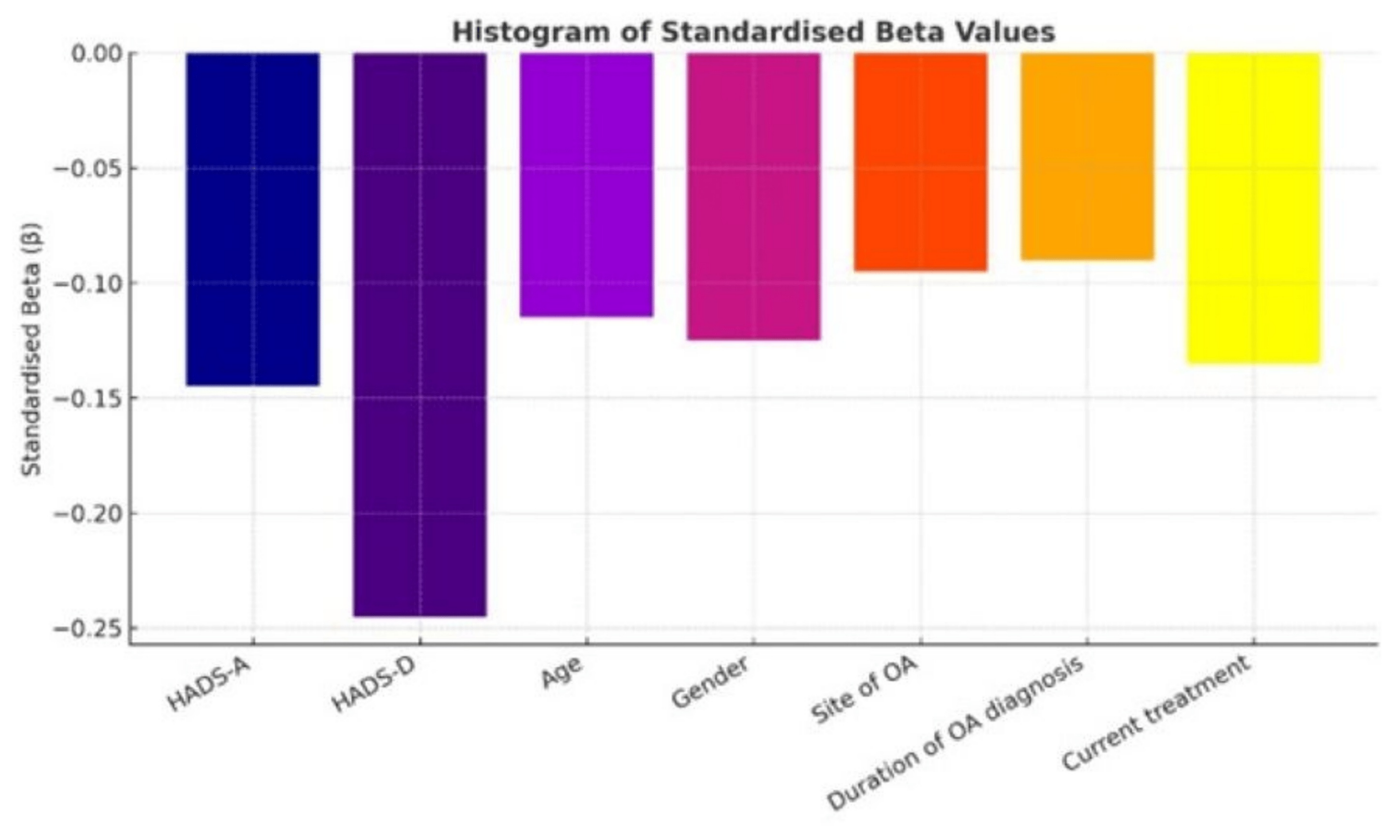
Distribution of Standardised Beta (β) Coefficients for Significant Predictors of Short Form Health Survey Scores in Patients with Osteoarthritis Standardised beta (β) coefficients for significant predictors of Short Form Health Survey [14] scores in patients with osteoarthritis. Predictors: Hospital Anxiety and Depression Scale–Anxiety [13], Hospital Anxiety and Depression Scale–Depression [13], age, gender, site of OA, duration of OA Diagnosis, and current treatment.

Figure 1 shows standardised beta (beta) coefficients of significant predictors that affect Short Form Health Survey scores in patients with OA. The outcomes revealed that depression (HADS-D) and anxiety (HADS-A) were the strongest negative influences on the HRQoL, with depression displaying the most significant effect (β ≈ 0.23), followed by anxiety (β ≈ 0.14). Age and gender also have a negative and less-strong influence (β ≈ -0.10 each). Consistently, the site of OA, duration of diagnosis, and current treatment are correlated with small but significant negative impacts on the health survey results. Psychological factors (most notably depression) seem to have a greater negative QoL impact on patients with OA than demographic and clinical factors.

Table 8 presents the distribution of OA locations and the management to which patients are currently subjected, among 300 patients. In total, 36 (12%) received no treatment, 74 (25%) took pain medications, 85 (28%) received intra-articular injections, 73 (24%) received physiotherapy, and 32 (11%) received surgery. There were site-specific patterns: hip OA was the most common, where 95 (32%) patients were managed with pain medications (45, 15%) and injections (28, 9%). Knee OA was present in 48 (16%) individuals, and a significant number of individuals had received injections (14, 5%). OA in the hand was present in 58 (19%), and the most common treatment for them was physiotherapy (22, 7%). Forty (13%) reported spine involvement, most being treated with injections (22, 7%) and physiotherapy (14, 5%). Less frequent sites of injury included the foot/ankle (27, 9%), shoulder (22, 7%), and elbow (10, 3%), with a wide variety of management strategies. A chi-square test indicated a statistically significant relationship between the site of OA and its current treatment (χ² = 137.74, df = 24, p < 0.001).

**Table 8:** Association Between the Site of Osteoarthritis and Current Treatment (N = 300) Data are presented as N (%); the chi-square test of independence was used to assess the relationship between the site of osteoarthritis and current treatment frequency; χ²(24, N = 300) = 137.74; statistical significance considered at p < 0.01**.

Osteoarthritis site	No treatment	Pain medications	Intra-articular injections	Physiotherapy	Surgery	Total	Chi-square χ² (df = 24)	p
Knee	19 (6%)	5 (2%)	14 (5%)	7 (2%)	3 (1%)	48 (16%)	-	-
Hip	5 (2%)	45 (15%)	28 (9%)	9 (3%)	8 (3%)	95 (32%)	-	-
Hand (fingers/thumb)	3 (1%)	19 (6%)	9 (3%)	22 (7%)	5 (2%)	58 (19%)	-	-
Foot/ankle	2 (1%)	2 (1%)	5 (2%)	9 (3%)	9 (3%)	27 (9%)	-	-
Shoulder	1 (1%)	2 (1%)	5 (2%)	9 (3%)	5 (2%)	22 (7%)	-	-
Elbow	2 (1%)	1 (1%)	2 (1%)	3 (1%)	2 (1%)	10 (3%)	-	-
Spine (neck/back)	4 (1%)	0 (0%)	22 (7%)	14 (5%)	0 (0%)	40 (13%)	-	-
Total	36 (12%)	74 (25%)	85 (28%)	73 (24%)	32 (11%)	300 (100%)	137.74	<0.001^**^

**Figure 2:**
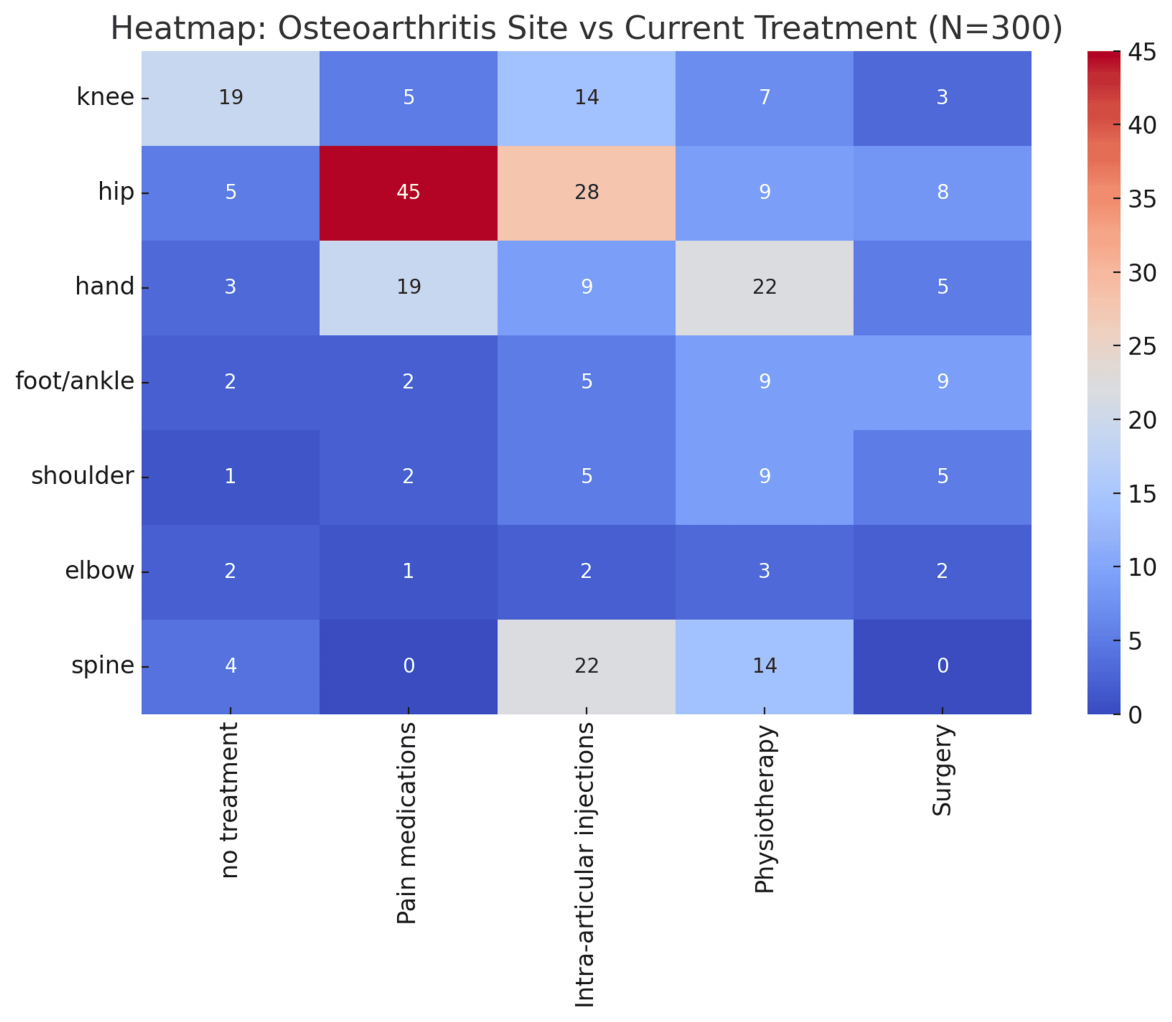
Heatmap of the Osteoarthritis Site by Current Treatment (N = 300) Warmer colours represent higher frequencies, while cooler colours represent lower frequencies. χ² (24, N = 300) = 137.74, p <0.001.

Figure 2 plots the distribution of OA sites along with the current treatments of 300 patients as a heatmap. Warmer colours represent increasing frequency, whereas lower frequencies are depicted through more fabulous shades. The hip joint has the closest associations with pain medications (n = 45), intra-articular injections (n = 28), and physiotherapy (n = 9). Abnormal treatment patterns are also evident in the spine, particularly with intra-articular injections (n = 22) and physiotherapy (n = 14). Knee OA is often treated with intra-articular injection (n=14) and pain medications (n=5), whereas OA of the hand is usually managed through physiotherapy (n=22) and pain medications (n=19). The foot/ankle, shoulder, and elbow are other sites with relatively low treatment frequencies across modalities. The heat map demonstrates high volatility in treatment approaches across different anatomical locations, with hip and spine cases being most intensely approached, followed by the spine and other sites (X² = 137.74, p < 0.001).

## Discussion

This research study was aimed at analysing the association of depression and anxiety with the QoL in Pakistan. In our study, the correlation between anxiety and depression was moderately positive, meaning that the more the participant is anxious, the more the chances of having depressive symptoms. This finding is consistent with the previous theoretical frameworks that also assume that highly anxious people are particularly vulnerable to the development of depression when exposed to stress and misfortunes [16]. We established that there was a negative correlation between anxiety and QoL, such that the higher the level of anxiety, the lower the QoL. This is corroborated by the latest literature that points out that the extent of anxiety is associated with reduced QoL and that functional impairment, inability to regulate emotions, and avoidant coping underlie the causal nature of anxiety [17]. The relationship between depression and QoL was negative in our study, as compared to anxiety. This is in line with the evidence of systematic reviews that state that depression is always associated with low QoL [18].

We found that the anxiety level significantly increased among female patients as compared to male patients. This finding is consistent with the data obtained in systematic reviews demonstrating that women are more predisposed to anxiety than men, which can be attributed to both psychosocial and biological factors [19]. We also found that female patients rated significantly higher in depression as compared to their male counterparts. This is in concurrence with previous research that suggests that women are more vulnerable to depression than men, and this is exhibited in adolescence. It persists in adulthood [20]. Our results have shown that male patients reported a significantly better QoL compared to female patients. This is consistent with the prior evidence of women having lower QoL than men, with depression as a factor in this difference [21].

We found that older adults scored higher on anxiety, which could be partially attributed to the fact that older people constituted a greater percentage of our sample. In contrast to a meta-analysis, which showed lower prevalence of specific phobia with age, our own use of a symptom-based scale (HADS-A) may have yielded subthreshold anxiety, which is still high in later life [22]. In our analysis, there were significant age-related increases in depression scores, with older adults reporting the highest scores compared to younger populations. This is consistent with a meta-analysis study across the world, which found a prevalence of 28.4% depression in older people, and, therefore, a heavy presence of depressive symptoms among the elderly in diverse populations [23]. In our analysis, the QoL decreased markedly with increasing age, with younger adulthood yielding higher scores and older adulthood yielding lower scores. Other cohort studies also provided similar results, with older patients having lower baseline QoL than younger ones [24].

Anxiety scores were not notably different among OA sites in our study, which indicated that joint location might not be a significant factor in determining anxiety levels. This result is consistent with past studies that found anxiety in patients with OA to have closer relations with pain severity than with disease location [25]. Depression scores varied significantly by OA site, with higher scores in elbow and spine OA compared to foot/ankle OA in our study. The same results were also found in a study, in which the role of depression was proven to be a significant contributor towards the severity of OA symptoms, especially among patients with mild-to-moderate disease [26]. Our findings indicated similar QoL among sites of OA, which indicated that disease location might not be a significant factor. This is consistent with a prior study, which identified pain severity and associated clinical factors as more predictive of worse QoL [27].

Our results are in line with a previous study, which also proved depression to have a strong relationship with poor QoL. This reaffirms that depression negatively impacts QoL across diverse populations, including patients with OA [18]. We also discovered that anxiety was negatively associated with poorer QoL, but its predictive strength was not as high as that of depression. The results of this finding are congruent with the recent reviews that found that increased anxiety symptom levels have correlated with poorer QoL. Some of the factors that lead to the correlation between anxiety symptoms and poor QoL include maladaptive coping, distress, and functional impairment [17]. We have found that older age is positively associated with lower QoL, which aligns with the existing literature that suggests older patients are more likely to report lower QoL scores. This finding highlights the vulnerability of the population under consideration [24]. We observed that the female participants scored lower on QoL than the male participants in our study, which is consistent with other studies that found that women typically scored low on QoL and high on depressive symptoms compared to men. This implies that the difference in genders in terms of QoL could be partly mediated by the high rates of depression in women [21]. We found that a poorer QoL was associated with both the site of OA and increased disease duration. An earlier study also found that increased joint involvement and disease duration substantially decreased HRQoL [27]. In our research, OA treatment was associated with a reduced QoL, indicating the severity of the disease. It is consistent with the prior studies that indicated that patients who have their hips or knees replaced initially report lower physical and social QoL, but the QoL is enhanced significantly post-surgery [28].

In our study, hip OA patients were more likely to undergo intra-articular injection, whereas hand OA patients tended to undergo physiotherapy. These results are consistent with other studies that have indicated that injections are helpful to manage pain in hip OA and that hydrotherapy or exercise is beneficial to manage pain, grip strength, and function in hand OA [29,30].

In the Pakistani setting, in which mental health care accessibility is still underdeveloped, the findings point to the necessity of developing culturally relevant interventions affecting physical and psychological aspects of OA.

Limitations

There are several limitations to this study that need to be considered when interpreting the findings. To begin with, the cross-sectional study does not allow for the development of causal relationships between anxiety, depression, and QoL in patients with OA. Second, the research used self-reported questionnaires (HADS and SF-36) as the only source, and it can be biased in terms of reporting, as well as failing to provide clinical diagnoses of psychiatric diseases. Third, the convenience sample used might restrict the ability to generalise findings to other areas of Pakistan and to patients with different socioeconomic or cultural backgrounds. Moreover, the HADS and SF-36 were administered in their original English versions without cultural or linguistic adaptation, which may have impacted measurement validity in a population with variable literacy levels. In addition, the study did not include OA and pain severity, as well as medication adherence, which may have contributed to the psychological results and QoL. Both of these contextual variables are crucial for understanding patient outcomes and should be taken into account in future research. Finally, the lack of longitudinal follow-up limits the evidence on how depression and anxiety may change over time, insofar as the progression of the disease and therapy are involved.

Suggestions for further investigation

Longitudinal studies should be conducted in the future to investigate the temporal variations of psychological variables and QoL in OA patients. Even more detailed information on predictors of psychological distress and functional impairment would be brought by the research that stratifies results based on the severity of the disease, the level of pain, and the method of treatment. To increase the credibility of mental health assessments, clinical psychiatric assessment should be added to the self-reports. Moreover, multicenter studies, which would include variable geographic and socioeconomic populations in Pakistan, would enhance generalisability. Intervention-based studies (mainly randomised controlled trials) investigating the combination of psychological and physical rehabilitation programs could provide insight into practical strategies to improve the QoL and mental health of patients with OA.

## Conclusions

This study highlights that both depression and anxiety are significantly associated with lower QoL in patients with OA in Pakistan, with depression showing a stronger association than anxiety. The female gender, old age, length of the disease, and specific locations of OA were also associated with worse outcomes. The research outcomes underscore the necessity of implementing an integrative approach to treating OA that emphasises both physical and psychological health. The routine care should include depression and anxiety screening and care to improve the patient's well-being and functional outcomes. By understanding the psychological impact of OA, medical care experts can develop more comprehensive treatment procedures that enhance the overall QoL for individuals with the condition. Nevertheless, given the cross-sectional design, the findings should be interpreted as associations rather than causal relationships.

## Appendices

**Table 9:** Demographic Information Questionnaire

Items	Responses
Age	-
Gender	-
Marital Status	-
Educational Level	-
Occupation	-
Site of osteoarthritis	-
Duration of osteoarthritis diagnosis	-
Comorbid conditions (you may select more than one)	-
Current treatment for osteoarthritis	-
